# Patient perspectives on factors influencing active surveillance adherence for low‐risk prostate cancer: A qualitative study

**DOI:** 10.1002/cam4.6847

**Published:** 2023-12-27

**Authors:** Lalita Subramanian, Sarah T. Hawley, Ted A. Skolarus, Aaron Rankin, Michael D. Fetters, Karla Witzke, Jason Chen, Archana Radhakrishnan

**Affiliations:** ^1^ Department of Internal Medicine University of Michigan Ann Arbor Michigan USA; ^2^ Center for Clinical Management Research, Health Services Research & Development VA Ann Arbor Healthcare System Ann Arbor Michigan USA; ^3^ Department of Surgery, Urology Section University of Chicago Chicago Illinois USA; ^4^ Department of Family Medicine University of Michigan Ann Arbor Michigan USA; ^5^ Department of Urology MyMichigan Health Midland Michigan USA

**Keywords:** active surveillance, primary care physicians, prostate cancer, quality of life, trust, urologists

## Abstract

**Background:**

Prostate cancer is the most common cancer among men in the United States. Treatment guidelines recommend active surveillance for low‐risk prostate cancer, which involves monitoring for progression, to avoid or delay definitive treatments and their side effects. Despite increased uptake, adherence to surveillance remains a challenge.

**Methods:**

We conducted semi‐structured, qualitative, virtual interviews based on the Theoretical Domains Framework (TDF), with men (15) who were or had been on active surveillance for their low‐risk prostate cancer in 2020. Interviews were transcribed and coded under TDF's behavioral theory‐based domains. We analyzed domains related to adherence to surveillance using constructivist grounded theory to identify themes influencing decision processes in adherence.

**Results:**

The TDF domains of emotion, beliefs about consequences, environmental context and resources, and social influences were most relevant to surveillance adherence‐. From these four TDF domains, three themes emerged as underlying decision processes: trust in surveillance as treatment, quality of life, and experiences of self and others. Positive perceptions of these three themes supported adherence while negative perceptions contributed to non‐adherence (i.e., not receiving follow‐up or stopping surveillance). The relationship between the TDF domains and themes provided a theoretical process describing factors impacting active surveillance adherence for men with low‐risk prostate cancer.

**Conclusions:**

Men identified key factors impacting active surveillance adherence that provide opportunities for clinical implementation and practice improvement. Future efforts should focus on multi‐level interventions that foster trust in surveillance as treatment, emphasize quality of life benefits and enhance patients' interpersonal experiences while on surveillance to optimize adherence.

## Graphical abstract

Decision processes related to staying on or stopping active surveillance for prostate cancer involved trust in active surveillance as treatment, quality of life and experiences of self and others. Positive perceptions of these three themes supported adherence while negative perceptions contributed to non‐adherence.

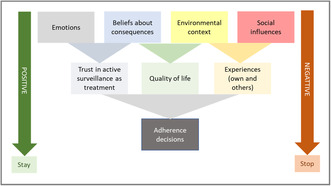

## 1 BACKGROUND

Prostate cancer is the most common cancer diagnosed in men in the United States. Nearly 200,000 men are newly diagnosed every year, resulting in more than 3 million men living with prostate cancer. This is in part due to increased awareness and screening for prostate cancer resulting in early detection.^1^ Given the typically slow‐growing nature of this cancer, guidelines have evolved over the past decade from definitive treatment to active surveillance as the preferred management strategy for low‐risk prostate cancer.^2^ Active surveillance avoids or delays treatments associated with considerable risk of side effects by regularly monitoring for disease progression. A combination of prostate‐specific antigen (PSA) tests, prostate imaging (e.g., magnetic resonance imaging), biopsies and rectal exams are part of routine care in active surveillance.^1^

Uptake of active surveillance as initial treatment has steadily increased; however, adherence remains a challenge.^3^, ^4^, ^5^, ^6^, ^7^, ^8^ The low overall adherence includes both men not receiving the recommended follow‐up and men deciding to stop active surveillance even when it remains clinically appropriate.^3^, ^9^ For example, in a statewide cohort of men with low‐risk prostate cancer, nearly half of the men on active surveillance did not receive the recommended follow‐up, with the majority not receiving their annual PSA testing.^10^ Others have shown that up to a third of men on active surveillance transition to receive surgery or radiation without evidence of cancer progression.^11^, ^12^ The reasons for non‐adherence remain unclear. Several studies have focused on decision‐making related to choosing active surveillance leading to improved uptake.^10^, ^13^, ^14^, ^15^, ^16^, ^17^, ^18^, ^19^, ^20^ Now, additional insights into factors influencing adherence to active surveillance are needed to similarly move the needle on mitigating non‐adherence.^21^, ^22^, ^23^, ^24^

To improve our understanding about adherence to active surveillance, we leveraged a statewide quality improvement collaborative to recruit men with low‐risk prostate cancer who were or had been on active surveillance to understand their perspectives on factors influencing adherence.^25^

## 2 METHODS

We conducted this study among men with low‐risk prostate cancer cared for in the Michigan Urological Surgery Improvement Collaborative (MUSIC) in 2020. MUSIC is a physician‐led, quality improvement collaborative in Michigan aimed at improving urologic care.^25^ It is comprised of 46 academic and community urology practices, and represents over 90% of the urologists across the state, thereby encompassing a diverse patient population.^26^ MUSIC has several initiatives aimed at optimizing active surveillance care delivery.

### 2.1 Conceptual framework and interview guide development

The interview guides were informed by the Theoretical Domains Framework (TDF), an implementation science framework^27^ comprised of domains based on psychological behavior change theories that can be used to characterize determinants influencing patient decisions related to active surveillance adherence. Importantly, TDF domains can be linked to the Behavior Change Wheel's Capability, Opportunity and Motivation (COM‐B) model,^28^, ^29^ to inform targeted intervention development in the future.^30^

The interview guide (see Data S1) assessed several aspects of active surveillance including: (1) knowledge (e.g., about active surveillance as a treatment choice and recommended follow‐up), (2) facilitators and barriers (i.e., determinants) to active surveillance adherence, and (3) preferences for provider roles in active surveillance care delivery. The guides were refined after pilot tests by the study team, including an urologist (TS), a primary care physician (PCP) (AR), an expert in cancer treatment decision‐making (SH), and two qualitative methodology experts (MF and DW).

### 2.2 Participants

Eligible participants included men with low‐risk prostate cancer who were or had been on active surveillance. Initially, emails were sent to MUSIC urologists asking them to identify eligible patients; however, recruitment became challenging due to the COVID‐19 pandemic. Therefore, recruitment was augmented in two ways. First, the study team requested permission from the providers who participated in this study to directly contact eligible patients in their practice (data from provider interviews is not discussed in this paper). Second, we posted the study on the University of Michigan research study website, which allowed interested patients to search and contact the study team. Eligible patients were enrolled after they provided informed consent. Participants were offered a $20 gift card for their time.

### 2.3 Data collection and analysis

Two members of the study team (AR and AJR) conducted individual, semi‐structured interviews with 15 patients. Due to the COVID‐19 pandemic, all interviews were conducted virtually over Zoom. All participants provided their informed consent at the start of their interviews. Interviews were audio recorded and professionally transcribed.

NVivo12 software was used to organize and facilitate analysis.^30^ All transcripts were deductively coded using the TDF domains and constructs organized in the COM‐B model as the basis of the coding scheme. First, two research team members (AJR, LS) independently mapped all interview content by line‐by‐line coding to a relevant TDF domain. This was done iteratively where after coding five interview transcripts, the study team met to review the coding scheme, refine coding definitions, and resolve any coding discrepancies. Second, the study team (AJR, LS, and AR) subsequently mapped all TDF domain content to constructs. Because of the overlapping nature of the domains in TDF, the same text was coded to more than one domain. For this study, the coding priority was given to the more frequently coded domain. Each domain was then reviewed to identify those that contributed the most to decisions regarding active surveillance adherence to shortlist key domains. Constructivist grounded theory was used from the perspective of the researcher reflecting on participant data and enquiring into underlying decision‐making processes. Reflecting on codes within each of the key domains from this perspective, and constant comparison within and across domains, enabled the identification of underlying themes that were then used to develop theory.^31^, ^32^ The qualitative analysis methods adhered to the consolidated criteria for reporting qualitative research (COREQ) checklist.^33^

### 2.4 Ethics approval

This study was reviewed and deemed exempt by the University of Michigan's Institutional Review Board (UM IRBMED HUM00175929).

## 3 RESULTS

Participants were, on average, 65 years old and White. The majority were married (80%) and had at least a college degree (87%). Men had been on active surveillance for an average of 5 years (Table 1).

**TABLE 1:** Study‐related attributes of patient participants at time of interview.

Participant	Age (years)	Marital status	Time since diagnosis (years)	Length of time on AS (years)	Stopped AS (Y/N)	Reason for stopping AS, if applicable
P01	68	M	6	6	N	n/a
P02	72	M	5	5	N	n/a
P03	69	D	2	2	N	n/a
P04	77	M	16	16	N	n/a
P05	62	M	3	3	N	n/a
P06	55	M	1	1	N	n/a
P07	40	D	3	1	Y	Prostatectomy due to progression
P08	66	M	3	3	N	n/a
P09	69	M	5	2	Y	Investigative treatment participant
P10	80	M	6	6	N	n/a
P11	54	M	1	1	N	n/a
P12	75	M	4	2	Y	Prostatectomy due to progression
P13	67	M	3	3	Y	Radiation therapy due to progression
P14	61	M	5	5	N	n/a
P15	77	D	3	3	N	n/a

Abbreviations: AS, active surveillance; D, divorced; M, married; N, no; n/a, not applicable; Y, yes.

**FIGURE 1:**
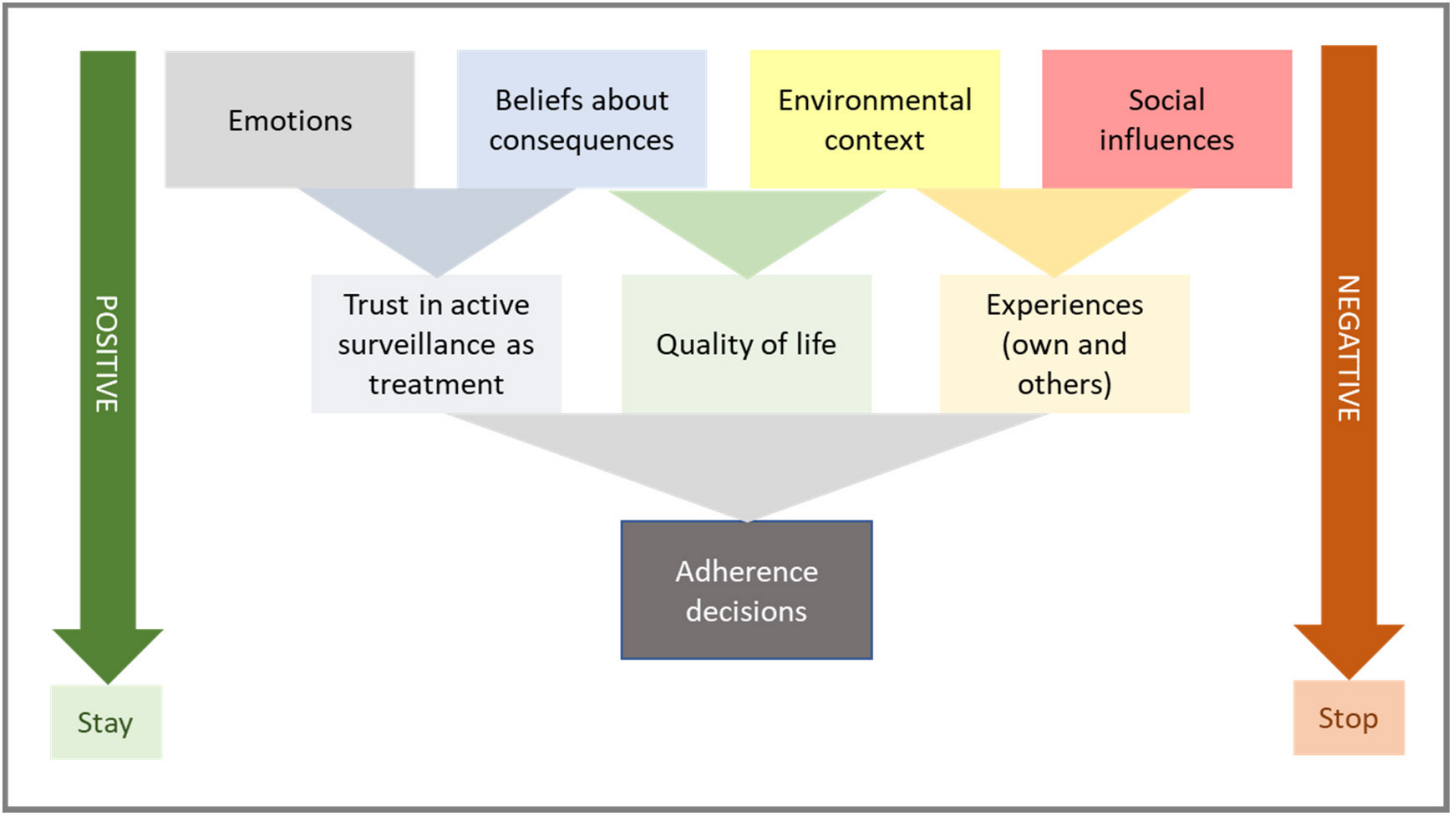
Behavioral theory‐informed conceptual model of adherence to active surveillance for men with low‐risk prostate cancer.

Men referenced the TDF domains of emotion, beliefs about consequences, environmental context and resources, and social influences as most relevant to their decisions regarding active surveillance adherence. From these key TDF domains, spanning motivation and opportunity in the COM‐B, using grounded theory, three underlying themes emerged: (a) trust in active surveillance as treatment, (b) impact of active surveillance on quality of life, and (c) influence of self and others' experiences on attitude towards active surveillance (Table 2). These codes were often double coded with the memory, attention, and decision processes TDF domain, supporting their influence on decisions related to adherence (Table 3). The emergent themes, the corresponding TDF domains and associated mapping to the COM‐B model are described below (Figure 1).

**TABLE 2:** Themes that emerged from TDF domains related to adherence to active surveillance.

Theme	Domain and definition	Quotes
Trust in active surveillance as treatment	Emotion A complex reaction pattern, involving experiential, behavioral, and physiological elements, by which the individual attempts to deal with a personally significant matter or event (adherence to active surveillance)	You do at times go what if it is, you know, it can be the silent killer, so to speak. But that's why it's important for me to say, okay, the PSA is staying the same, my symptoms are staying the same
		we've got there and you see it's probably the lowest level and it's being controlled and it's being monitored by people you trust actually there is a bit of relief that goes with that.
		No, actually I probably feel more comfortable that I'm under care and it's staying contained, so there's actually a bit of relief to that than living with it and not knowing it was going to explode and if you get it how bad it's going to be and are you going to have to have surgery right away?Am I going to be wearing a diaper? All this stuff.
		As for low‐risk I mean I, if you maybe start with the premise that maybe everybody has some cancerous cells in their body that their body is capable of keeping under control. If that's your starting point then this is not a very eventful situation. If you think of it as a black or white situation of absolutely having no possibility of any cancer cells in your body, then it would be more alarming. But I guess my feelings are more that it's more of a severity thing than a black or white, yes or no diagnosis
	Beliefs about consequences Acceptance of the truth, reality, or validity about outcomes of a behavior in a given situation (adherence to active surveillance)	Because in my mind it tells me I don't need anything – any surgery or radiation performed immediately, I can wait. I can wait, if ever having it done. It may stay, I may stay as the intermediate or lower intermediate forever.
		you kind of get used to it and if things don't change very much, you just figure, okay. They're probably right. No, it's not getting any worse
		It's a problem that we can address and depending on what happens, this might be the kind of prostate cancer that never changes and most men will end up dying with and not from, but dying with never knowing they had it. And that's kind of, okay, I can live with that
Quality of life	Beliefs about consequences	To me, the physical sexual component was a huge issue for me, just because I'm so young, and you know. If I had been 65, 70, and I was married to the same woman for 50 years, I mean, yeah, it would suck, and it would be terrible, but you know, we kind of had been there, done that sort of thing.
		yeah, I didn't like the side effects at all actually on the surgery…Well, it could be ED, which didn't thrill me at all, that was the big one for me anyway. That's the game changer for me.
		The side effects are so severe compared to the potential of growth, it was like, we read like 10% or 20% chance of growing in over a ten‐year span, I think. And 60% to 80% chance of the side effects, so, yeah, that was scary.
		So I, in particular, noticed that with the surgery and understanding the nerves, the number of nerves that are there, the fact that this surgery could have some really uncomfortable side effects or results of the surgery, I wasn't terribly interested in that. Kind of considered the radiation, the localized with the little whatever that is…Little beads, yeah. And, again, kind of considered that, just tried to learn as much as possible and with everything pointing towards this being an early stage, I'm in reasonably good health.
		Truly, my prostate is exactly a non‐issue for me from a day‐to‐day perspective. It doesn't cause me pain, doesn't cause me any issues that I can tell. It's something that I have to think about because it's got cancer, but beyond that, I don't notice it
Quality of life	Environmental context and resources Any circumstance of a person's situation or environment (adherence to active surveillance) that discourages or encourages the development of skills and abilities, independence, social competence and adaptive behavior	For good or for bad I probably haven't changed my habits much. I drink alcohol some and fatty foods. Nothing crazy. We've never been one to eat badly, I don't think, but at the same time I kind of made that conscious choice to say, well, I could do some things that might improve my chances a bit. But the alternative to living a lifestyle that's not something I look forward to day to day or what, however to phrase that, I just kind of go about my normal life at this point.
Experience while on active surveillance	Environmental context and resources	The last one [biopsy] was not nearly as much fun. Maybe it's because the area is getting more sensitive, I have no idea. for one thing I couldn't see what was going on…It was decidedly more painful.
		The one [biopsy] at xxx was really painful and like I knew I had to do it. Like a year later I knew I had to do it, but it was definitely something that I was dreading.
		Well, I don't know, maybe one would be, I guess a little more preparedness for that first initial, whatever, which one, that rectal test. Yeah, I kind of went in there thinking, a biopsy, I've had it done let's say on skin, that's what I'm thinking. And what it did was put a real huge fear into me. Even today when I think of it…
		And I discovered it, right after I retired from the fire department. Had I discovered it four months earlier, then everything would have been covered under the Cancer Presumption Act, which they passed for firefighters, which included the prostate…. All the financial part of it could have been covered, but they don't do it retroactively. So, I'm on my own, and my insurance is adequate, but it's not good, I end up paying a lot for it. So, there's some financial stress involved in it too. Especially now that I'm on a fixed income
Experiences of family and peers	Social Influences Those interpersonal processes that can cause individuals to change their thoughts, feelings, or behavior (about adhering to active surveillance)	And I had a friend of mine, a guy I worked with, he's a few years old than me, and he, his prostate kind of swelled up, and he went through that and had to do the hot core thing and was on a catheter for a month. I said, “Well, I don't want to do that either.” So I figured, before it gets to the point where I've got to put up with all these other, not necessarily inconveniences, I'll just get it out
		And they found that there, well, one of the small sections of it. And then the next year, went back and had another biopsy. And by then, it had grown a little bit more. And we made the decision at that point to do the prostatectomy and get it out, which is something that my father advised me a long time ago because that's what he did. And that, you figure that saved his life, and I think it probably did the same for me.

**TABLE 3:** Interaction of active surveillance adherence themes with decision making. Sample quotes describing overlap of positive and negative aspects of themes with decisions related to adherence and staying or stopping active surveillance.

Theme	Sample quotes	
	Positive—supports adherence and staying on active surveillance	Negative—contributes to leaving or non‐adherence to active surveillance
Trust in active surveillance as treatment	I'm very comfortable that with the help of the doctors we're keeping a very close eye on it. I'm feeling very comfortable with treatment.	I can say that I'm a little nervous doing the active surveillance, but just thinking that we are missing an opportunity, that it is very small, that it could become more severe in the next six months if it shows a big growth
	I guess just being educated on the pros and cons of each different option, that's what keeps me on active surveillance.	
	I trusted the doctors, I trusted the test. It was made fairly clear to me that this seemed to be a rather small location, it turned out to be bigger than they thought. But the test showed fairly small and fairly slow growing. Because again, the PSA wasn't going like this, it was up, it was elevated, but it wasn't going by leaps and bounds	
Quality of life	I am not anxious to have something done if I don't have to	And I had a friend of mine, a guy I worked with, he's a few years old than me, and he, his prostate kind of swelled up, and he went through that and had to do the hot core thing and was on a catheter for a month. I said, “Well, I don't want to do that either.” So I figured, before it gets to the point where I've got to put up with all these other, not necessarily inconveniences, I'll just get it out
	there was talk from physicians at xxx, that sometimes you go ahead and get treatment simply for peace of mind. Okay, and that was not my goal. My goal was to postpone treatment the maximum amount of time I could without putting severe health at risk of negative outcomes	
	And for me it's not 80. Quite frankly, if I make it ten years I don't care. I have a limited amount of time with my son and during that time I both want to be active and alive and worried about him and playing with him and doing stuff with him. Not focused on recovery from a surgery.	
Experiences	You know, I had friends who had worse conditions than mine. So I would hear from them about what they were going through and it was clear to me that mine was almost insignificant in comparison to them. That made a big difference to me by the way, knowing about other people and how bad they were. They were getting Gleason 7 and Gleason 8 kinds of diagnoses and were very concerned and they've had significant operations since those assessments and I've had nothing	if you had somebody who died from it maybe, maybe if my father had died from prostate cancer I might have thought different. I might have said, “I'm not going to wait around three or four or five years. It's inevitable we're going to have to operate eventually. In my mind I might as well do it when I'm younger rather than older, easier to recover from it and all that stuff.” All the reasons you'd rather have the operation sooner rather than later

### 3.1 Trust in active surveillance as treatment

Trust in active surveillance as treatment emerged as a theme from two TDF domains: *Emotion* and *Beliefs about Consequences*. Men discussed whether they trusted the medical evidence that recommended surveillance was the right treatment option for them and recognized that surveillance involved a protocol of labs, imaging, and biopsies to monitor cancer progression. Trust in the treatment facilitated adherence.

#### 3.1.1 Emotion [COM‐B motivation]

The TDF defines *Emotion* as a complex reaction pattern, involving experiential, behavioral, and physiological elements, by which the individual attempts to deal with a personally significant matter or event. Men reflected on the presence or absence of anxiety impacting their decision to adhere to surveillance. The follow‐up required on surveillance both contributed to worry and provided reassurance. Some men discussed the anxiety they felt prior to their routine testing, where the results may suggest cancer progression. But routine testing was also reassuring since it monitored for cancer progression. As one patient commented, *You do at times go, what if it is, you know, it can be the silent killer…But that's why it's important for me to say, okay, the PSA is staying the same, my symptoms are staying the same* [68 years, 6 years on active surveillance].

Those who trusted in the medical evidence for the appropriateness of surveillance were more comfortable in their treatment choice and less anxious, which in turn promoted adherence. Some men commented that, in fact, it was possible for them to not give much thought to their cancer diagnosis between follow‐up tests.

as far as my worry, I am concerned and cognizant of it [disease progression], I think about it. but I don't let it wear me down every single day. I'm not walking on eggshells; I'm living my life

[61 years, 5 years on active surveillance].

#### 3.1.2 Beliefs about consequences [COM‐B motivation]

*Beliefs about consequences* relates to an acceptance of the truth, reality, or validity about outcomes of a behavior in a given situation. Trust in active surveillance follow‐up protocols promoted the belief that negative consequences of cancer and definitive treatment could be delayed, and helped men adhere to surveillance. For example, one patient said, *Because in my mind it [active surveillance] tells me I don't need anything – any surgery or radiation performed immediately, I can wait. I can wait, if ever having it done. It may stay, I may stay as the intermediate or lower intermediate forever* [69 years, 2 years on active surveillance]. Meanwhile, the absence of this trust caused worry about potential negative consequences of uncontrolled cancer leading to transitioning to definitive treatment.

### 3.2 Quality of life

Quality of life emerged as a theme from two TDF domains: *Beliefs about consequences* and *Environmental context*. Decision‐making regarding active surveillance adherence were influenced either by the direct impact of surveillance on quality of life, or indirectly, where the alternative treatment choice was perceived as more likely to significantly affect one's quality of life.

#### 3.2.1 Beliefs about consequences [COM‐B motivation]

Men's beliefs about the consequences of treatment options for their low‐risk prostate cancer and the subsequent impact on their quality of life influenced decisions related to adherence. Men commonly discussed the potential detrimental side effects (consequences) of radiation and surgery, such as bowel and urinary incontinence and erectile dysfunction. These symptoms were viewed to negatively impact quality of life and as such, were a major consideration in promoting adherence to surveillance. As one patient noted, *the side effects are so severe compared to the potential of growth…that was scary* [55 years, 1 year on active surveillance].

While several patients described how active surveillance allowed them to avoid side effects and thus, maximize their quality of life, some believed that definitive treatment was an eventuality. Men factored in potential challenges with recovering from surgery or radiation at an older age in deciding whether to stay on or opt out of active surveillance. A poor or lengthy recovery would then negatively impact their subsequent quality of life. This concept was elucidated in this quote: *I'm not going to wait around three or four or five years. It's inevitable we're going to have to operate eventually. In my mind I might as well do it when I'm younger rather than older, easier to recover from it and all that stuff. All the reasons you'd rather have the operation sooner rather than later* [72 years, 5 years on active surveillance].

#### 3.2.2 Environmental context [COM‐B opportunity]

Environmental context relates to any circumstance of a person's situation or environment that discourages or encourages the development of skills and abilities, independence, social competence and adaptive behavior. In this study, environmental context was applied to any aspect of active surveillance that was a feature of this treatment option or context of care, outside of the patient's behavior or agency. The environmental context included barriers to and facilitators of active surveillance, such as pain from routine biopsies or quality of care.

The absence of drastic interventions, often involving side effects, periods of recovery, and hospitalization, was a feature of active surveillance that caused the least perturbation in men's quality of life, and therefore viewed positively. This impacted men's decisions to adhere to surveillance. A participant who had opted to adhere to surveillance said, *Truly, my prostate is exactly a non‐issue for me from a day‐to‐day perspective. It doesn't cause me pain, doesn't cause me any issues that I can tell. It's something that I have to think about because it's got cancer, but beyond that, I don't notice it* [54 age, 1 year on active surveillance].

### 3.3 Experiences of self and others

Experiences emerged as a theme from the TDF domains of environmental context (person‐environment interaction) and social influences. An individual's own experiences with their care and those of others within their social circles influenced decisions related to active surveillance adherence. Notably, negative experiences had substantial influence on non‐adherence.

#### 3.3.1 Environmental context and resources [COM‐B Opportunity]

A common barrier that nearly all the men mentioned were the painful repeat prostate biopsies they had to undergo on surveillance. Men reported not being prepared adequately by their providers in terms of what to expect during and after the procedure. This negative experience had the potential to impact their adherence as men commented on considering not returning for follow‐up biopsies. *The one [biopsy] at XXX was really painful and like I knew I had to do it. Like a year later I knew I had to do it, but it was definitely something that I was dreading* [62 years, 3 years on active surveillance].

#### 3.3.2 Social influences [COM‐B opportunity]

Social influences are those interpersonal processes that can cause individuals to change their thoughts, feelings, or behaviors. It comprises the constructs of social norms and social support. Men reflected on the active surveillance experiences of their immediate family members and peers. For example, one participant commented, *And we made the decision at that point to do the prostatectomy and get it out, which is something that my father advised me a long time ago because that's what he did. And that, you figure that saved his life, and I think it probably did the same for me* [75 age, 2 years on active surveillance]. Another chose to adhere to active surveillance when he learned about his friends' experiences and said, *I had friends who had worse conditions than mine. So, I would hear from them about what they were going through and it was clear to me that mine was almost insignificant in comparison to them* [80 years, 6 years on active surveillance].

Men also commented on the absence of peer support which had the potential to negatively impact surveillance adherence. Support groups were viewed to be beneficial: they allowed men to discuss factors impacting their decisions related to active surveillance with other men who had/were experiencing the same treatment. They could discuss the uncertainty of being on surveillance and the risk of cancer progression and whether remaining on surveillance was the right choice. Men commented on the sensitive nature of symptoms and consequences of prostate cancer treatment like urinary incontinence or erectile dysfunction and felt they would benefit from sharing experiences with other men diagnosed with prostate cancer. As noted by a man, *Now these are all embarrassing things to kind of talk about on some level, but I think over the years, it's gotten easier just because it's, you know, it's a part of my life now, you know. But I would say, the more people that, like myself, who can be advocates or people that can talk to people that are going through this, I think is because you can only get it from somebody who's been through it…* [40 years, 1 years on active surveillance].

## 4 DISCUSSION

This study used a behavioral theory‐based framework, the TDF, to identify patient‐level determinants contributing to adherence to active surveillance for men with low‐risk prostate cancer. The rise in guideline‐based adoption of active surveillance as primary treatment now demands meaningful support for sustained participation and adherence from patients throughout their management period. Patients identified four key domains contributing to adherence: *Emotions, Beliefs About Consequences, Environmental Context*, and S*ocial Influences*. These in turn contributed to patients' trust in active surveillance for treatment, assessing the impact of surveillance on quality of life, and influencing their attitudes towards active surveillance adherence. While favorable perceptions promoted adherence, the lack of trust, uncertainties related to outcomes and negative experiences on surveillance, factored into non‐adherence, including transitioning to definitive treatment options.

Trust in active surveillance as treatment reduced anxiety and facilitated adherence for men in our study. This is important as prior research has shown that men on active surveillance often have prostate‐specific anxiety while on treatment and that anxiety and uncertainty related to disease progression negatively impacted mental well‐being.^34^, ^35^, ^36^ Several modifiable and non‐modifiable factors have been shown to impact decisions related to active surveillance adherence.^37^ This study now provides additional support for a modifiable factor, that is ensuring men understand what active surveillance is and what follow‐up entails to successfully monitor for cancer progression.^38^ An informed patient is motivated to adhere to surveillance which in turn generates trust in the treatment and can regulate negative emotions such as anxiety (*Motivation*). In fact, in recent calls for patient‐focused psychosocial support to promote adherence to active surveillance, the importance of men understanding their disease was key.^23^, ^39^

Quality of life was a key driver for surveillance adherence. Prior studies have shown that sexual and general physical function have been low among those who had opted for definitive treatments.^34^, ^35^ Thus, emphasis on quality of life benefits of active surveillance could help promote its adherence.

A qualitative study of men who had dropped out of active surveillance at a single care site in London, UK, identified similar, negative factors associated with stopping active surveillance.^22^ These included poor experience at diagnosis and follow‐up, inconsistent communication and lack of support from the care team, partners and peers. Despite an inability to interview men who had dropped out from active surveillance, the importance of support and trust in active surveillance through better, consistent communication and follow‐up were identified in this study as important for promoting adherence.

Consistent with prior work,^22^ this study also identified that negative personal experiences contribute to non‐adherence. In addition, this study identified that men are also influenced by the experiences of others, either positively or negatively. Therefore, presenting men with the *Opportunity* to engage with their peers not only remedies isolation but also provides a venue to calibrate norms based on others' experiences. For example, support groups could educate and prepare men for painful biopsies, provide venues to air concerns about sensitive topics like sexual health and urinary function, and share experiences.

By combining constructivist grounded theory and the TDF framework, this study uncovered the relationship between beliefs about consequences, nature of the treatment (environmental context), and quality of life. Aspects of these factors have been identified by others in different healthcare contexts and populations suggesting that the identified themes are not unique to the men interviewed in this study.^21^, ^22^, ^23^, ^24^ The significant and novel contribution made by this study is in uncovering the interaction between these factors and proposing how they might influence decision making related to continuing or stopping active surveillance, summarized in Figure 1.

This study has some limitations. First, the COVID‐19 pandemic did not allow for in‐person recruitment or interviews. Virtual discussions could have made interviews feel more impersonal. Additionally, access to technology and internet proved to be a requirement for study participation and thereby could have introduced unavoidable selection bias. Due to the nature of qualitative research, other potential biases may also exist (e.g., a primary care doctor conducting the interviews may have induced social desirability bias in participant responses). However, rigorous processes were followed and clearly outlined in the methods, to ensure reliability and validity. Second, despite oversampling for men of racial/ethnic minority and those who had transitioned off active surveillance during recruitment, the participant diversity was limited. The study was unable to recruit patients who had disengaged with active surveillance follow‐up or opted out without clinical evidence of disease progression. Nevertheless, participants described issues with their care that provided insights into challenges with adherence that were aligned with prior literature.^22^, ^24^, ^37^, ^38^, ^39^ Further, by achieving thematic saturation from our interviews with men spanning different ages, different lengths of time on surveillance and receiving care in different settings, this rich data still enabled the identification of important mechanisms underlying adherence decision processes. Future studies in other geographic, cultural, and healthcare contexts might provide additional insights on patient‐level factors impacting adherence.

## 5 CONCLUSION

This study identified the relationship between *Beliefs About Consequences, Emotions, Environmental Context*, *and Resources* and *Social Influences* on underlying themes of trust in active surveillance as treatment, quality of life and experiences (of self and others) that influence adherence to active surveillance for men with low‐risk prostate cancer. Understanding this relationship can inform the development and implementation of interventions in the future to support patients on active surveillance to maximize adherence.

## AUTHOR CONTRIBUTIONS

**Lalita Subramanian:** Conceptualization (equal); formal analysis (equal); methodology (equal); visualization (equal); writing – original draft (equal); writing – review and editing (equal). **Sarah T. Hawley:** Conceptualization (supporting); formal analysis (supporting); writing – review and editing (equal). **Ted A. Skolarus:** Conceptualization (supporting); formal analysis (supporting); methodology (supporting); writing – review and editing (equal). **Aaron Rankin:** Data curation (lead); formal analysis (equal); writing – review and editing (equal). **Michael D. Fetters:** Conceptualization (supporting); formal analysis (supporting); methodology (supporting). **Karla Witzke:** Writing – review and editing (equal). **Jason Chen:** Writing – review and editing (equal). **Archana Radhakrishnan:** Conceptualization (lead); data curation (lead); formal analysis (supporting); funding acquisition (lead); investigation (lead); methodology (lead); project administration (lead); resources (lead); supervision (lead); writing – original draft (supporting); writing – review and editing (lead).

## CONFLICT OF INTEREST STATEMENT

The authors declare that they have no competing interests.

## Supporting information


Data S1:

## Data Availability

The data that support the findings of this study are available from the corresponding author, Archana Radhakrishnan, upon reasonable request.
