# Supplementary material for: Patient perspectives on factors influencing active surveillance adherence for low‐risk prostate cancer: A qualitative study
Source: Cancer Med. 2023 Dec 27;13(1):e6847. doi: 10.1002/cam4.6847 (PMC10807559; doi:10.1002/cam4.6847)
Supplement: Supplementary file 1 — Data S1: [file CAM4-13-e6847-s001.pdf]

<u>Appendix 1</u>

Patient and Partner Interview Guide

Date:
Interviewer:
Participant Name:
Practice Name:

Key issues to address during Interview:

Attitudes/Believes of Patient/Partner regarding Active Surveillance
Treatment Decision Process / Continuing Decision Process
Partner Role in Decision Process

Notes for Interviewer:

Conduct the interview with both the patient and partner at the same time. If a theme comes up that might be better addressed separately, you can say the following:

> "Now, sometimes with some issues like dealing with medical problems, we may have private thoughts or feelings that influence our decisions. Things you may or may not feel are even important, but these kinds of information are important to our research. In order to facilitate that kind of discussion, I'd like to take a few minutes to speak with each of you alone, would that be ok?"

Introduction:

"First off, I'd like to thank you for agreeing to an interview today. May name is __________, and I am from the University of Michigan. This study is funded by the National Cancer Institute. Our goal is to understand how we take care of men with low-risk prostate cancer. As a patient with a low-risk prostate cancer, we're really interested in your views and experiences. We will also be interviewing other patients and doctors in Michigan.

Purpose and Agenda:

"During this interview today we will be talking about your experiences with your prostate cancer. I am most interested in what you went through and thought about regarding the issues we discuss, so please don't feel shy, your views are very valuable to us, and we are here to learn from you. I have a list of topics I would like to discuss, but feel free to bring up any topics you feel are related to our discussion. I want to let you know that this interview is completely voluntary, so if you want to stop at any time or don't feel comfortable answering a question please let me know."

Recording:

"I would like to record our discussion so that we can review your views exactly and we don't miss out on anything you say. Our discussion will remain completely confidential, only the research team will listen to the recording and the information you give will only be used for this research project. Is it OK to record the discussion?"

Duration:

"Every interview is different and so this may go anywhere from 30mins to over an hour. Do you have any questions?"

Ice-Breaker:

Since we're talking about your prostate today, it'd be helpful for me to know who all you see to get care for your prostate?
-can probe if they saw physicians before that they don't see know (for example, spoke with radiation oncologist at time of diagnosis)

What is your understanding about the condition of your prostate right now?

- How does patient refer to their "cancer"?
- Understand if patient does have cancer. Probe their understanding of the severity/risk of their cancer.
- Use their term for the rest of the interview.

What is the approach to your treatment of your XXXuse the term patient usedXXX right now?

- If the patient is describing everything having to do with active surveillance but not using the term, okay to probe if they've heard of the term "active surveillance" being used to describe their treatment.

~~One of the reasons we wanted to talk to you is because your doctor told us you are on active surveillance for your prostate cancer, is that what you call it?~~

What does active surveillance mean to you?

- In the medical world, we mean following men with low-risk prostate cancer closely with bloodwork, studies to take images of your prostate, and tests that look at the cells of the tissue of your prostate using biopsy.
- **Come to agreement with interviewee on what language to use moving forward.**

Pay attention to what they call AS, PCP, Prostate Condition etc, and reflect this back later in the interview.

I want you take me through your journey, in detail, from when you were diagnosed with [low-risk prostate cancer] to now.

- Can you tell me a little bit about how you were feeling when your PSA came back elevated and you were diagnosed with prostate cancer?
- Has how you felt changed since your diagnosis? What are you feeling now?

Intentions/ Goals/ Memory, attention, and decision processes /Social influences/Beliefs about consequences

What makes you stay on (or what made you drop out of) [AS]?

- Reflecting on your time experience, can you talk about things that would help someone stay on [AS]?
- Did/Do your doctors play any role in this decision?
    - Which doctors?
- What are the things that you/your partner do/does to make you stay on or drop out of [AS]?
    - Tell me about a recent example.

Emotion/ Behavioral regulation

We know that some men worry about their XXXprostate cancerXXX progressing while being on [AS].

- What has been your experience with this?
- How (and when) do you talk about this with your doctors?
- How have you (or your providers) managed your worry?
- How (and when) do you talk about this with your partner/family?

Reinforcement/Environmental context and resources/Social influences

We know that sometimes there are others (like partners) who can play a role in men's treatment decisions for their [low-risk prostate cancer]. Can you both share your experience with each other when it comes to the treatment decision making for (your or your partner's) cancer?

Professional role and identity

What do you think the role specifically is for [PCPs] in managing your [AS]?

What do you think the role specifically is for urologists in managing your [AS]?

[PCPs] can and do work with urologists in taking care of their prostate cancer patient. Can you share with me your experience of how your [PCP] has worked with your urologist?

We're nearing towards the end of our interview. I just have a couple of questions left. If you were to design an intervention to help your patients, like you, with XXXlow-risk prostate cancerXXX stay on [AS], what would you focus on?

Before we end, is there anything else you think I ought to know about taking care of men with low-risk prostate cancer?
